# The Positive Effect of Moderate-Intensity Exercise on the Mirror Neuron System: An fNIRS Study

**DOI:** 10.3389/fpsyg.2019.00986

**Published:** 2019-05-03

**Authors:** Zebo Xu, Min Hu, Zi-Rong Wang, Jin Li, Xiao-Hui Hou, Ming-Qiang Xiang

**Affiliations:** ^1^ Department of Sports and Health, Guangzhou Sport University, Guangzhou, China; ^2^ Guangzhou Sport University, Guangzhou, China; ^3^ Department of Graduation, Guangzhou Sport University, Guangzhou, China

**Keywords:** mirror neuron system, social interaction, motor cognition, moderate-intensity exercise, fNIRS

## Abstract

A growing number of studies have reported the beneficial effect of exercise on human social behavior. The mirror neuron system (MNS) plays a critical role in a variety of social behaviors from imitation to empathy. However, neuroimaging investigations into the effects of exercise on the MNS remain unexplored. To address this question, our study determined the effect of moderate-intensity exercise on the MNS using functional near-infrared spectroscopy (fNIRS). Specifically, 23 right-handed young individuals were asked to perform a table-setting task that included action execution and action observation before and after a 25-min exercise session on a cycle ergometer at moderate intensity (65% VO_2peak_). The control condition was the same task performed without exercise. Cortical hemodynamic changes in the four primary brain regions of the MNS were monitored with fNIRS, using a modified probe configuration that covered all four MNS regions in the left hemisphere. We used a region of interest (ROI)-based group analysis to determine which regions were activated during action execution and action observation. Following a session of moderate-intensity exercise, we found a significant increase in activation in all four MNS regions, namely the inferior frontal gyrus (IFG), premotor cortex (PMC), superior parietal lobule (SPL), and rostral inferior parietal lobule (IPL). This result indicated a positive effect of exercise on the MNS, specifically that moderate-intensity exercise could activate the MNS.

## Introduction

In the past decade, there have been many studies investigating the effect of exercise on the brain. There is increasing evidence showing that exercise is beneficial for cognitive performance (Hillman et al., 2003; Yanagisawa et al., 2010), improves memory acquisition (Winter et al., 2007), prevents cognitive dysfunction in Parkinson’s disease (David et al., 2015), and improves social behavior in adolescents with Attention-Deficit/Hyperactivity Disorder (ADHD) (Kamp et al., 2014) and in children with autism (Pan, 2010). Social cognition is a complex process, and understanding its neural basis to improve its performance is essential for our survival. Human social behaviors in the brain are mainly controlled by the amygdala, orbitofrontal cortex (OFC), and the mirror neuron system (MNS) regions (Rizzolatti et al., 2001; Adolphs, 2003). Van Rensburg et al. (2009) proposed that exercise can have a positive effect on the OFC. However, the effect of exercise on the MNS remains in question.

Rizzolatti et al. (1996) were first to discover the MNS; it fired when a macaque monkey grasped food (action execution) as well as when the monkey observed the experimenter grasping food (action observation). The MNS responsible for these behaviors was discovered in area F5 and in area PF and PFG of the rostral inferior parietal lobule (IPL) in non-human primates (Rizzolatti et al., 1996; Rizzolatti and Craighero, 2004; Fogassi et al., 2005). Research has also ascertained the brain regions that make up the human MNS; the human homolog of area F5 contains the premotor cortex [PMC; Brodmann area (BA) 6] and the inferior frontal gyrus (IFG; BA44/45), and the most likely homolog for area PF/PFG is located in the rostral part of the human IPL (BA40) and the superior parietal lobule (SPL; BA7) (Buccino et al., 2001; Rizzolatti and Craighero, 2004; Filimon et al., 2007; Kilner et al., 2009; Molenberghs et al., 2010). In terms of human social cognition, the MNS has been reported to control the ability to understand the actions of others (Rizzolatti et al., 2001), to imitate (Iacoboni et al., 1999; Rizzolatti and Craighero, 2004), to communicate using gestures and speech (Rizzolatti and Arbib, 1998; Rizzolatti and Craighero, 2004) and to act in cooperation with others (Egetemeir et al., 2011). Therefore, the basic functions of the MNS include matching the other person’s action in our mind, and when we next observe a similar action, the MNS would be activated again. The MNS represents the neural network that helps people identify their own actions and those of others, in order to understand the purpose of others’ actions by observation (Gallese et al., 2004), which we refer to as action understanding. In our study, we used both action observation and action execution to determine whether exercise has a positive effect on the function of the MNS. If we had only used action execution to reflect the activation of MNS, it would have been difficult to parse out whether the MNS activation was caused by action understanding or actual physical movement.

Although many neuroimaging studies have investigated the MNS with fMRI and positron emission tomography (PET) (Grafton et al., 1996; Moriguchi et al., 2009), these techniques are restricted to a constrained measuring environment to avoid off-target brain activation. In contrast, functional near-infrared spectroscopy (fNIRS) is more portable, allowing subjects to perform tasks in a natural and comfortable environment. This eliminates the confounding factor of delay between the variable being studied and brain imaging to assess activation. Sun and the colleagues established fNIRS as a non-invasive technique to detect the activation of MNS brain regions during action execution and observation (Sun et al., 2018). Accordingly, fNIRS has become an ideal tool to explore the MNS.

In this study, we used fNIRS to investigate whether activation of the MNS regions in the human brain changed after exercise. This is the first exploration of the possible effects of moderate-intensity exercise on the four dominant regions of the MNS, namely the IFG (BA44/45), PMC (BA6), rostral IPL (BA40), and SPL (BA7), which together cover almost all the mirror neuron-related regions in the brain.

## Materials and Methods

### Subjects

We recruited 23 healthy subjects (12 males and 11 females) with a mean age of 20.7 ± 1.6 years (range 18–25) to participate in this study. The average body weight of the study cohort was 65.2 ± 11.7 kg and the average height was 169.5 ± 9.0 cm. All subjects were right-handed according to the Edinburgh Handedness Questionnaire (Oldfield, 1971) and had normal or corrected-to-normal vision. None of the subjects had a history of neurological, major medical, or psychiatric disorders, and none were taking medication at the time of the fNIRS measurement. All subjects participated in three sessions (training, experimental, and control sessions) and were instructed to avoid strenuous exercise 24 h prior to each session. The study was carried out in accordance with the recommendations from the Ethic Committee of Guangzhou Sport University with written informed consent from all participants. All participants gave written informed consent in accordance with the Declaration of Helsinki. The protocol was approved by the Ethic Committee of Guangzhou Sport University.

### Experimental Design

**Figure 1:**
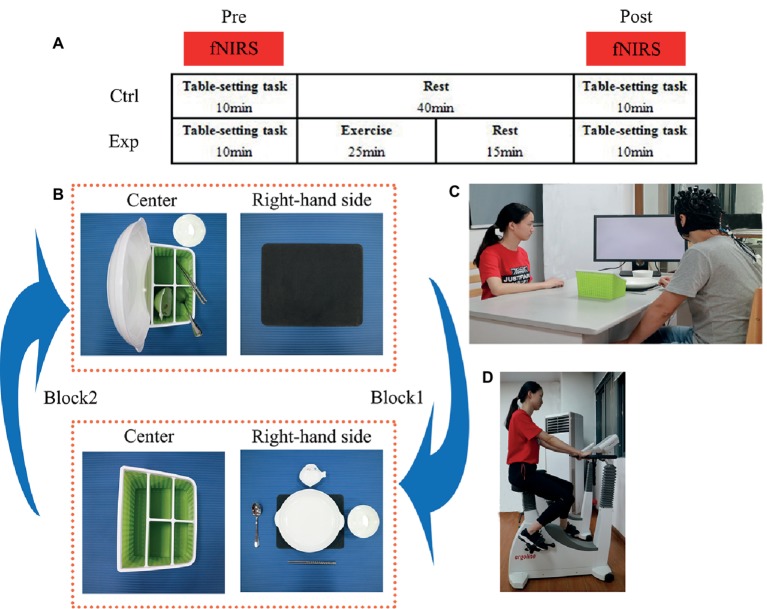
**(A)** The experimental design showing the experimental (exp) condition with moderate-intensity exercise and the control (ctrl) condition with no exercise (rest). Cortical hemodynamic changes were monitored with functional near-infrared spectroscopy (fNIRS) while subjects performed the table-setting task. The task was performed before (pre) and after (post) exercise or rest. **(B)** Illustration of the block design of the table-setting task. In block 1, the subject (in the action execution component) or the experimenter (in the action observation component) moved the five tableware items from the green storage box in the center to the black placemat on the right-hand side. In block 2, the five tableware items were moved back from the placemat to their original positions in the green storage box. **(C)** Brain activity was measured (black NIRSport cap with probes for fNIRS measurements) while subjects performed the table-setting task (informed written consent was obtained from for the publication of these images). **(D)** The experimenter illustrates the moderate-intensity exercise on the cycle ergometer (informed written consent was obtained from for the publication of these images).

During their first visit to our laboratory, subjects underwent a training session where they were familiarized with action execution and action observation until they were familiar with the rules and could adequately complete the entire task. Based on the classification of physical activity intensity of the American College of Sports Medicine (Medicine, 2013), the maximal oxygen uptake (VO_2peak_) was measured (MasterScreen CPX, CareFusion, Hoechberg, Germany) to determine the appropriate individual intensity for a moderate level of exercise. In the second and third sessions, the experimental (exp) and control (ctrl) protocols were conducted with a counterbalanced design on different days. Under the exp protocol, subjects conducted the action execution and observation components of the task before (pre) and after (post) a moderate-intensity level of exercise on the cycle ergometer. Under the ctrl protocol, subjects conducted the same action execution and observation components but rested instead of performing the exercise (Figure 1A).

### Table-Setting Task Preparation

We adopted a table-setting task to test action execution and observation. The subject and the experimenter sat face to face at a table. In the center of the table, a placemat was positioned on the right-hand side and a storage box of tableware in front. The tableware consisted of five objects: a plate, a saucer, a pair of chopsticks, a soupspoon, and a rice bowl. For the table-setting task, the subjects had to move the five tableware items from the storage box to the placemat on their right-hand side (and back from the placemat to the box) with their right hand at a normal, natural speed. A monitor was placed at a 45° angle in front of the subject to present visual cues: a cross to denote periods of rest and a picture of cups to denote the start of the task. During these rest periods, the subjects were asked to look at the cross on the monitor and avoid any action.

### Table-Setting Task Procedure

In the action execution (exec) component of the experiment, each subject was asked to look at the monitor for the appropriate visual cues. The cross was displayed for 20 s (rest period), followed by the picture of cups to begin the task. The exec component of the experiment was comprised of eight blocks, where block 1 consisted of 15 s to move the five tableware items to the placemat and 20 s to rest. In block 2, each subject returned the tableware items to their original positions in the storage box and then rested again for 20 s (Figures 1B,C). Block 3, block 5, and block 7 were the same as block 1; block 4, block 6, and block 8 were the same as block 2. The order of placement was always fixed: plate, saucer, chopsticks, soupspoon, and a rice bowl.

In the action observation (obs) component of the experiment, the monitor was turned toward the experimenter, and the same visual cues were displayed as in the exec component. The obs component also consisted of the same eight blocks. While the experimenter executed the actions, the subjects watched carefully, and when the experimenter stopped, the subjects rested. The subjects were instructed to remain still while carefully watching the actions of the experimenter and focusing on their right hand moving the tableware. The order of the two components of the task (exec and obs) was counterbalanced across subjects. In total, there were 16 blocks in exec and obs components.

An experimenter monitored the action of each subject throughout the experiment (all exec and obs components of the exp and ctrl protocols). All subjects reported that it was very easy to follow the experimenter’s actions in the obs component. Only one of the subjects felt slightly uncomfortable during the task, but it did not affect their ability to complete the task.

### Cardiovascular Exercise Test

As mentioned above, the exec and obs components of the table-setting task were performed during both the exp and ctrl sessions. During the exp session, subjects performed 25 min of exercise on a cycle ergometer (Ergoselect 100, ergoline GmbH, Germany) that consisted of a 5-in warm-up, 15 min of exercise at moderate intensity (65% VO_2peak_), and a 5 min recovery period (Figure 1D). A wireless heart-rate monitor (Acentas pulse meter, BM-CS5EU, Beijing, China) monitored the subject’s heart rate (HR) during the entire exercise period. The pedaling rate was maintained at 60 rpm. The resistance workload started out at 30 W and automatically increased in the last 2 min of the warm-up period till the HR monitor reached the target HR at 65% HR_peak_. Once the subject reached the target HR, the cycle ergometer computer decreased the resistance level to make sure each subject was exercising at moderate intensity for the 15-min exercise period. Finally, the cycle ergometer computer decreased the resistance level back to 30 W to let the subject cool down during the recovery period.

### fNIRS Data Acquisition

**Figure 2:**
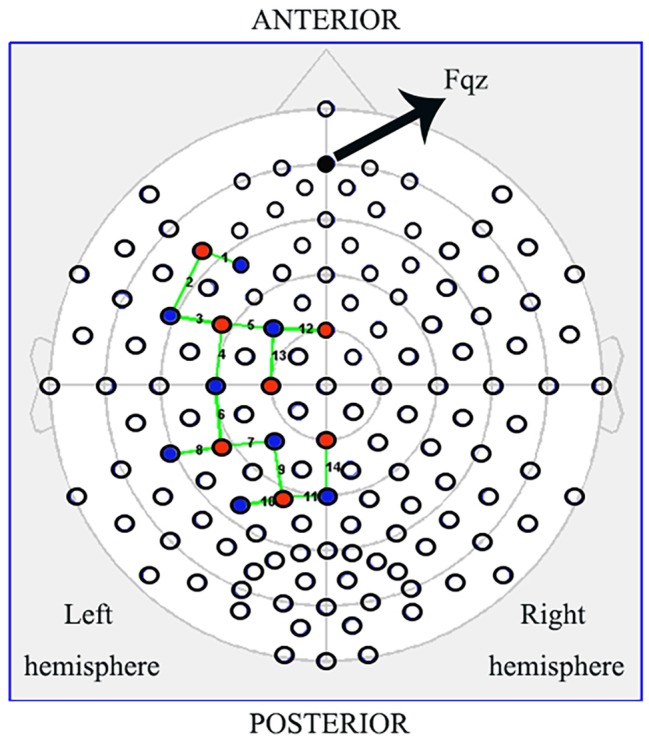
Schematic of the probe setup when positioned on the subject’s head. The NIRSport (NIRx Medical Technologies, LLC, Los) is a continuous-wave fNIRS system consisting of seven light sources (red) and eight detectors (blue) with a 3-cm source-detector separation, comprising 14 channels (green) across the IFG (BA44/45), PMC (BA6), rostral IPL (BA40), and SPL (BA7). Active probes were only in the left hemisphere.

fNIRS data were collected using the NIRSport mobile fNIRS system (NIRx Medical Technologies, LLC, New York, USA). This continuous-wave fNIRS system consisted of eight light sources and eight detectors, with 3-cm source-detector separations that formed of 14 channels. We recorded hemodynamic responses in the IFG (BA44/45), PMC (BA6), rostral IPL (BA40), and SPL (BA7), which together covered almost all mirror neuron-related regions in the left hemisphere. We placed active probes only in the left hemisphere because the left hemisphere is dominant when subjects perform a right-hand action (Figure 2, red dots = sources, blue = detectors; Filimon et al., 2007; Egetemeir et al., 2011). Each LED light source emitted light at two wavelengths (760 and 850 nm), and data were recorded at 7.81 Hz. The cap was positioned on the head by centering the bottom of the probe at the Fpz position (Figure 2), according to the international 10/20 positioning system (Zimeo Morais et al., 2018). In addition to the acquisition of hemodynamic response data during the table-setting task (pre/post, ctrl/exp, exec/obs), the baseline data were recorded, while each participant was resting and still for 15 s prior to the start of the table-setting test to avoid irrelevant activation (Fu et al., 2016).

### fNIRS Data Preprocessing

The NIRStar acquisition software (NIRx Medical Technologies, LLC, New York, USA) was used to record fNIRS data and to evaluate its signal-to-noise ratio. The nirsLAB data analysis package (NIRx Medical Technologies, LLC, New York, USA) was used for all subsequent calculations. Raw data for all channels were visually inspected, spike artifacts were removed, and faulty channels were removed from subsequent analyses. All channels were band-pass filtered, with a low-cutoff frequency of 0.01 Hz and a high-cutoff frequency of 0.1 Hz to remove baseline drift and physiological noise, respectively (Kirilina et al., 2013). The modified Beer-Lambert law was used to compute estimates of changes in oxygenated hemoglobin (OxyHb), deoxygenated hemoglobin (DeoxyHb), and total hemoglobin (totalHb) levels from the frequency filtered data (Sassaroli and Fantini, 2004).

### Statistical Analyses

The statistical parametric mapping (SPM) utilities incorporated into nirsLAB were used to determine event-related changes in OxyHb, DeoxyHb, and totalHb during executed and observed actions. SPM employed the general linear model (GLM) to identify OxyHb, DeoxyHb, and totalHb hemodynamic brain responses with reference to experimental factors. First-level analyses (SPM 1) assessed differences on a within-session basis, and second-level analyses (SPM 2) assessed differences on a between-session basis. IBM SPSS Statistics 22 was used to analyze the OxyHb beta-value for each subject, condition, and channel obtained in SPM 1.

The traditional channel-based group analysis was used to assess the activation pattern. Two sides, one-sample *t*-tests were performed based on the individual-level beta-values, to determine if those channels significantly activated during the exec component compared with rest period were also activated in the obs component (*p* < 0.05, FDR-corrected). To achieve better spatial consistency, a region of interest (ROI)-based group analysis was also implemented to assess the activation pattern using two sides, one-sample *t*-tests.

In order to examine the effect of moderate-intensity exercise on the MNS, we first investigated the four MNS ROIs in response to both action execution and observation during the presessions of both the exp and ctrl conditions, which were free from any effects of exercise or rest periods. Then, each ROI was analyzed using a repeated-measures ANOVA with condition (exp/ctrl) and session (pre/post) as within-subject factors. The exp and ctrl results were compared with a paired *t*-test.

## Results

### Activation Pattern Assessed Using Channel-Based Group Analysis

First, we analyzed channel-by-channel to explore whether those channels activated during the exec component were also activated in the obs component after exercise. The beta-values of OxyHb for each task were compared to the corresponding baseline (15 s prior to table-setting task onset) OxyHb. When acquiring fNIRS data, changes in OxyHb and DeoxyHb concentration were measured simultaneously. However, there is some scientific disagreement regarding which signal to use to analyze brain activation. In our study, we mainly focused on the OxyHb signal because it was often observed to have higher amplitude than the DeoxyHb signal (Strangman et al., 2002; Yanagisawa et al., 2010). In other words, the signal-to-noise ratio of OxyHb is better, and this signal is more sensitive to task response (Cheng et al., 2015).

The results showed that 11 channels were significantly activated during both exec and obs components (*p* < 0.05, FDR-corrected) in the post-exp condition, which is the exercise-intervention condition. Activated channels were located over all the ROIs, which are IFG (BA44/45), PMC (BA6), rostral IPL (BA40), and SPL (BA7). Moreover, there were five channels strongly activated in the post-exp-obs condition (channel 2, 6, 9, 11, and 13; *p* < 0.01, FDR-corrected), located over the IFG (BA44/45), PMC (BA6), and SPL (BA7).

**Figure 3:**
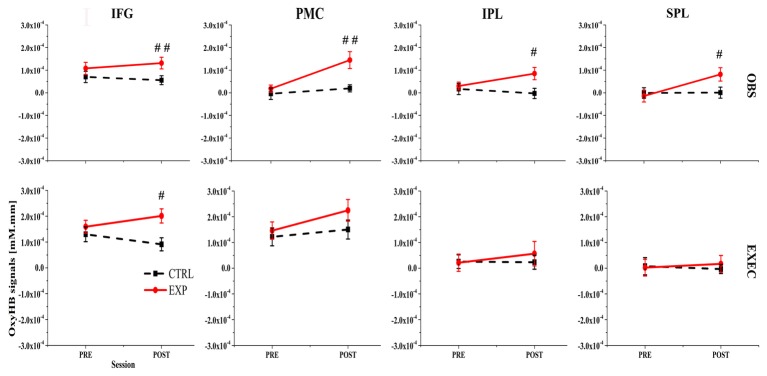
First row lists the action observation (OBS) component and the second row lists the action execution (EXEC) component. IFG, inferior frontal gyrus; PMC, premotor cortex; SPL, superior parietal lobule; IPL, inferior parietal lobule; # represents a significant difference between pre-ctrl and pre-exp condition or post-ctrl and post-exp condition, #*p* < 0.05; ##*p* < 0.01; ###*p* < 0.001.

Next, we compared action execution and observation in the no-exercise condition, which were free from any effect of exercise. Ten channels were activated during both action execution and action observation in the post-ctrl condition (channel 3, 4, 5, 6, 7, 9, 11, 12, 13, and 14; *p* < 0.05, FDR-corrected), which covered all of the brain regions. According to the spatial map of the 23 subjects in the pre-exp condition and pre-ctrl condition, we also found that channels 2, 3, 4, 5, 6, 7, 9, 10, and 13, which mainly belong to IFG (BA44/45), PMC (BA6), and rostral IPL (BA40), were activated during both action execution and observation (*p* < 0.05, FDR-corrected). Figure 3 illustrates the activated level channel-by-channel in both conditions (*p* < 0.05, uncorrected).

### Activation Pattern Assessed Using ROI-Based Group Analysis

Channel-based group analysis of our data showed inconsistent channel activation between the post-ctrl and pre-exp conditions, which despite being two separate groups, still represented the same experimental condition. In order to achieve better spatial consistency, an ROI-based group analysis was implemented (Yanagisawa et al., 2010). Specific regions of interest were selected: the left IFG (channels 1, 2, and 3), the left PMC (channels 4, 5, 12, and 13), the left rostral IPL (channels 6, 7, and 8), and the left SPL (channels 9, 10, 11, and 14). We analyzed them individually to determine which ROIs were activated during action execution and action observation in the exp and ctrl conditions.

We first used ROI-based group analysis to determine which brain area was activated after exercise. We found that four brain regions, namely the IFG (BA44/45), PMC (BA6), SPL (BA7), and the rostral IPL (BA40) were significantly activated during action observation and execution in the post-exp condition (*p* < 0.05, FDR-corrected) except SPL (BA7) during action execution.

The ROI-based analysis results also showed that nearly all of the brain regions, the IFG (BA44/45), PMC (BA6), rostral IPL (BA40), and SPL (BA7) were significantly activated during action observation and execution in all the no-exercise conditions (*p* < 0.05, FDR-corrected), which included the post-ctrl, pre-exp, and pre-ctrl conditions. Only the activation of the SPL (BA7) during action execution in the post-ctrl condition and the rostral IPL (BA40) during action execution in the post-ctrl condition were not significantly activated (*p* > 0.05, uncorrected).

### ROI-Based Group Analysis for the Effect of Moderate-Intensity Exercise

We used a paired *t-*test to statistically compare the cortical activation pattern assessed by ROI-based group analysis in the pre-exp, pre-ctrl, and post-ctrl conditions, which were free from any effects of exercise. As expected, there were no significant differences in any ROIs during action execution or action observation between pre-exp, pre-ctrl, and post-exp conditions (*p* > 0.05, FDR-corrected).

During action observation, the ANOVAs for three of the four ROIs revealed marginally significant interactions between the condition (exp/ctrl) and session (pre/post) factors. Specifically, the ANOVA results were as follows: the IFG (BA44/45) resulted in *F*(1, 22) = 2.64, *p* = 0.118, *η*^2^ = 0.107; the PMC (BA6) showed *F*(1, 22) = 4.73, *p* = 0.041, *η*^2^ = 0.177; and the rostral IPL (BA40) was calculated as *F*(1, 22) = 3.52, *p* = 0.074, *η*^2^ = 0.138. Then we used a paired *t*-test to statistically compare the cortical activation pattern between presessions (exp/ctrl) or postsessions (exp/ctrl). During the presessions, there were no significant differences between the exp and ctrl conditions (*p* > 0.05, FDR-corrected). However, during the postsessions, there were significant differences between the exp and ctrl conditions in all four ROIs (*p* < 0.05, FDR-corrected).

In contrast, during action execution, only the OxyHb signal in the left IFG (BA44/45) was significantly greater in the post-exp condition compared with the post-ctrl condition (*p* < 0.05, FDR-corrected). These results demonstrated that moderate exercise led to increased Oxy-Hb-related cortical activation in the left IFG (BA44/45), PMC (BA6), rostral IPL (BA40), and SPL (BA7). Figure 3 presents the OxyHb signal change for both conditions across all ROIs.

**Figure 4:**
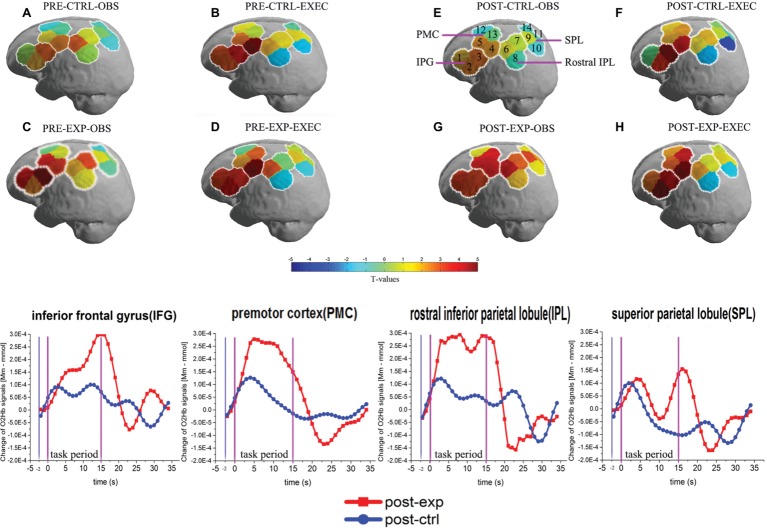
Activation patterns of the MNS after exercise when using the ROI-based group analysis. MNS brain regions activated in the left hemisphere during action observation prior to exercise or rest **(A,C)**, and action execution prior to exercise or rest **(B,D)**, action observation following exercise or rest **(E,G)**, action execution following exercise or rest **(F,H)**. OBS, action observation; EXEC, action execution; EXP, experimental condition/exercise; CTRL, control condition/rest; PRE, prior to EXP/CTRL; POST, following EXP/CTRL. Each small circle in the different colors represents one channel, and the color scale represents the t-values at the group level. Each white circle represents an ROI, namely the IFG (BA44/45), PMC (BA6), rostral IPL (BA40), and SPL (BA7). Representative graphs of the OxyHb signal change over time are shown in the bottom row of the figure, comparing action observation in the post-exp (red line) and post-ctrl (blue line) conditions in the four ROIs. OxyHb signals are shown in arbitrary units (mM mm). Baseline OxyHb level (2 s before task onset, denoted by the first two vertical lines) is also shown.

In order to intuitively observe the changes in cortical activation by task, we presented Figure 4, and the graphs in the lower row show the representative graph of OxyHb change along the experimental timeframe for all the ROIs during action observation in the post-exp condition and post-ctrl condition. In each graph, we can clearly see the increased hemodynamic response in the post-exp condition compared with the post-ctrl condition. The raw data supporting the conclusion of this manuscript are available upon request to authors.

## Discussion

### Detecting the Activation of the MNS Using fNIRS

Our study adopted a modified task and probe configuration of fNIRS to investigate the effect of moderate-intensity exercise on the MNS. We focused on the four dominant brain regions of the MNS, namely the IFG (BA44/45), PMC (BA6), SPL (BA7), and rostral IPL (BA40), and were able to detect changes in the activity of all four regions when using ROI-based group analysis. These results proved that it is feasible to use fNIRS to investigate MNS activity, a finding corroborated by a previous study (Sun et al., 2018). Sun et al. (2018) also used fNIRS to measure changes in the MNS; however, the fNIRS parameters used in our study were different. Even without their double density probe configuration and a spatial resolution of 3 cm (compared with their spatial resolution of 1.5 cm), we could still detect MNS activity during both action execution and observation before and after moderate-intensity exercise.

### MNS Activation Increased After Moderate-Intensity Exercise

There are many studies that have provided evidence of the positive effect of exercise in children and adolescents with autism spectrum disorder (ASD), who have deficits in cognitive processing, impaired social interactions, delayed or limited communication skills, as well as confined activity patterns and interests. Exercise can reduce stereotypic behaviors and improve positive social skills (Magnusson et al., 2012; Olin et al., 2017). Short-duration exercise has been shown to improve performance in cognition tasks; the better the hemodynamic response, the better the cognitive performance (Yanagisawa et al., 2010; Lucas et al., 2012; Byun et al., 2014). Moreover, many studies have indicated that ASD brains have structural abnormalities in the MNS regions (Theoret et al., 2005; Hadjikhani et al., 2006) and that the MNS plays a critical role in social cognition (Gallese et al., 2004). It was reported that the MNS became activated when humans performed tasks related to social behaviors such as imitation, comprehension of intention, and emotional understanding (Iacoboni and Dapretto, 2006). However, there were no reports on whether exercise can have a positive effect on the MNS. We showed that OxyHb in some regions of the MNS was significantly elevated in subjects during action observation after moderate-intensity exercise. In addition, the social cognition task of table setting expects us to determine a sequence of action, a type of recall that requires activation of the IFG. We found that the activation of the IFG (BA44/45) was significantly increased during action execution after moderate-intensity exercise. Collectively, these results suggest that exercise leads to increased activation of the healthy MNS while performing and observing social cognition-related tasks. Although our study subjects were normal adolescents and young adults between 18 and 25 years of age, the data from our experiments can still contribute to our understanding of MNS dysfunctions in children with ASD or schizophrenia.

Mori and colleagues used fNIRS and proton magnetic resonance spectroscopy (H-MRS) to investigate the MNS, the left amygdala, and the bilateral orbitofrontal cortex (OFC) in children with autism. They found that the concentration of OxyHb in the pars opercularis of the IFG (BA44) in children with autism while they imitated emotional facial expressions was significantly lower than that of normal children. However, concentrations of OxyHb in this same area became significantly elevated in autism after they were trained to imitate emotional facial expressions (Mori et al., 2015). This study suggests that although dysfunction in the MNS may be one critical neurological feature of ASD, mirror neurons can be activated by repeated imitation in children with ASD. Similar to repeated imitation, our study also revealed moderate-intensity exercise to be another method to increase OxyHb in MNS brain regions while observing an action.

Many studies that investigated social behavior have reported a suppression of the MNS in ASD and schizophrenia. The MNS was not as activated in cases with ASD and schizophrenia compared with the healthy group (Iacoboni and Dapretto, 2006; Mier et al., 2010; Derntl and Habel, 2011). EEG data of action observation in ASD showed reduced mu rhythm suppression compared with the healthy group (Oberman et al., 2005). Dapretto and colleagues used fMRI to show that children with ASD exhibited less activation in the IFG compared with controls while observing and imitating emotional expressions (Dapretto et al., 2006). Moreover, transcranial magnetic stimulation (TMS) data showed reduced corticospinal facilitation during action observation in children with autism (Theoret et al., 2005; Habel et al., 2010). Using different paradigms and research techniques, these studies all indicated one common point—individuals with ASD and schizophrenia have impairments in their MNS brain regions. Several intervention studies impressively demonstrated that social cognitive deficits like repetitive behaviors are modifiable through specific sports training programs (Staples et al., 2011; Movahedi et al., 2013). An increasing body of literature supports that appropriate exercise can improve social cognitive deficits in individuals with disorders that include MNS dysfunction such as ASD and schizophrenia. Accordingly, we can assume that physical exercise activates certain brain regions that control social behaviors. Our data revealed a positive effect of moderate-intensity exercise on the MNS of healthy individuals; observing an action increased the hemodynamic activation of the MNS brain regions. This result suggests that the brain regions activated by exercise to improve social behavior in ASD could potentially include the MNS. Therefore, our findings put forward the idea that MNS activation could be a fundamental mechanism underlying the beneficial effects of exercise on social behaviors. In line with this idea, some studies have suggested that MNS activity may serve as a biomarker of psychiatric disorders if future research is able to conclusively associate social cognitive deficits with certain clinical symptoms and syndromes, and if the assessment of MNS dysfunction can be specifically linked to clinical outcome variables (Iacoboni and Dapretto, 2006; Derntl and Habel, 2011). Research specifically testing individuals with ASD and schizophrenia is needed to validate MNS activation as a mechanism linking exercise with improved social cognition and to investigate the potential of MNS activity as a diagnostic marker for such diseases.

### Does Exercise Improve Social Cognitive Behaviors by Direct MNS Activation or by Modifying Sensory Perception to Activate the MNS?

Exercise can modify our visual, auditory, and olfactory sensory perception by increasing blood flow or inducing other physical changes in the relevant brain regions (Delp et al., 2001; Krishnamurti and Grandjean, 2003; Davranche et al., 2005; Marioni et al., 2010; Si et al., 2011). Numerous studies have indicated that different exercise patterns have a positive effect on human sensory perception. Sensory perception deficits are some of the core symptoms in children with autism with MNS dysfunction (Robertson and Baron-Cohen, 2017). There are also many studies reporting that different types of exercise can have a positive effect on social cognition in children with autism; for example, aquatic exercises significantly improve the social interactions of children with autism (Yilmaz et al., 2004; Pan, 2010), and walking or jogging can reduce stereotypic behaviors (Prupas and Reid, 2001). From the knowledge gained so far in the field, can we speculate that exercise improves social performance by directly enhancing the function of the MNS? Or does exercise modify sensory perception, which changes our outlook toward the world and activates the MNS, leading to better social behaviors in our daily life? More research is needed to parse out the mechanism by which exercise-induced MNS activation improves social cognitive function.

## Limitations

One limitation of our study was the inconsistent activations we observed following the channel-based group analysis of the same condition (pre-ctrl, pre-exp, and post-ctrl). However, this can be explained by individual differences in brain shape and size. Channel-based group analysis assumes that the probes are positioned in the exact same locations in every subject; however, due to the different shapes of the head across different subjects, channel-based group analysis sometimes generates inconsistent activation results. Another limitation of this study was the weak activation of the rostral IPL (BA40) and SPL (BA7) in action execution and observation. This brain region was the weakest of the four key regions, which are not activated during action execution in the post-ctrl condition. It is possible that the limited number of probes was insufficient to optimally cover the rostral IPL (BA40) and SPL (BA7), preventing the activation of this brain region from being fully detected. A higher sample size and better probe placement could address this issue.

## Conclusion

Our study demonstrated that an individualized exercise program is an effective method to enhance hemodynamic responses in the MNS of healthy individuals. Our findings could present MNS activation as a potential mechanism for the beneficial effects of exercise on social integration in adolescents with ASD. The benefits of using exercise as an intervention include its cost-effectiveness and potentially preventative nature compared to other behavioral interventions. Thus, our study may also support the use of exercise as an intervention to improve social behavior in autistic and schizophrenic individuals *via* its positive effects on the function of the MNS.

## Ethics Statement

The study was carried out in accordance with the recommendations from the Ethic Committee of Guangzhou Sport University with written informed consent from all participants. All participants gave written informed consent in accordance with the Declaration of Helsinki. The protocol was approved by the Ethic Committee of Guangzhou Sport University.

## Author Contributions

ZX, MH, X-HH, and M-QX contributed to conception and design of the study. ZX, Z-RW, and JL organized the database. ZX and M-QX analyzed the data. ZX wrote the first draft of the manuscript. MH, X-HH, and M-QX contributed to manuscript revision, and read and approved the submitted version.

### Conflict of Interest Statement

The authors declare that the research was conducted in the absence of any commercial or financial relationships that could be construed as a potential conflict of interest.

## Abbreviations

BA: Brodmann area
ctrl: Control
exec: Action execution
exp: Experimental
fNIRS: Functional near-infrared spectroscopy
IFL: Inferior frontal gyrus
IPL: Rostral inferior parietal lobule
MNS: Mirror neuron system
obs: Action observation
PMC: Premotor cortex
post: Post
pre: Prior to
SPL: Superior parietal lobule
